# Giant Posterior Mediastinal Ancient Schwannoma Requiring Thoracoabdominal Resection: A Case Report and Literature Review

**DOI:** 10.4021/wjon348w

**Published:** 2011-08-24

**Authors:** Benjamin Quartey, Jeffrey Lenert, Subrato J Deb, Leonard R Henry

**Affiliations:** a National Capital Consortium, National Naval Medical Center, Department of General Surgery, Bethesda, Maryland, 20889, USA; b National Capital Consortium, National Naval Medical Center, Department of Surgical Oncology, Bethesda, Maryland, 20889, USA; c National Naval Medical Center, Department of Cardiothoracic Surgery, Bethesda, Maryland, 20889, USA; d Western Maryland Regional Medical Center, Thoracic Surgery Oncology, Cumberland, Maryland, 21502, USA; e Indiana University Health, Goshen Center for Cancer, Goshen, IN 46526, USA

**Keywords:** Giant, Schwannomas, Posterior mediastinum, Thoraco-abdominal

## Abstract

Posterior mediastinal schwannomas are benign, slow growing nerve sheath tumors and rarely cause symptoms. We present a case of a 47-year-old man who presents with severe mid-back pain and dyspnea on exertion. Chest radiograph and computed topography revealed a large posterior mediastinum mass. Surgical resection required en bloc resection of a portion of the diaphragm, and wedge resection of the left lower lobe of the lung via left thoracoabdominal approach. Pathology was consistent with ancient schwannoma. This case is unique due to the location and size of the mass and the surgical approach required for complete resection.

## Introduction

Schwannomas are nerve sheath tumors which commonly originate from the extremities, head, neck and the posterior mediastinum [1]. Most are benign, slow growing but can become massive in size causing significant local symptoms if not resected. We report a case of a giant left posterior mediastinal schwannoma causing back pain and dyspnea that required resection via thoracoabdominal approach due to location and involvement with surrounding structures.

## Case Report

**Figure 1:**
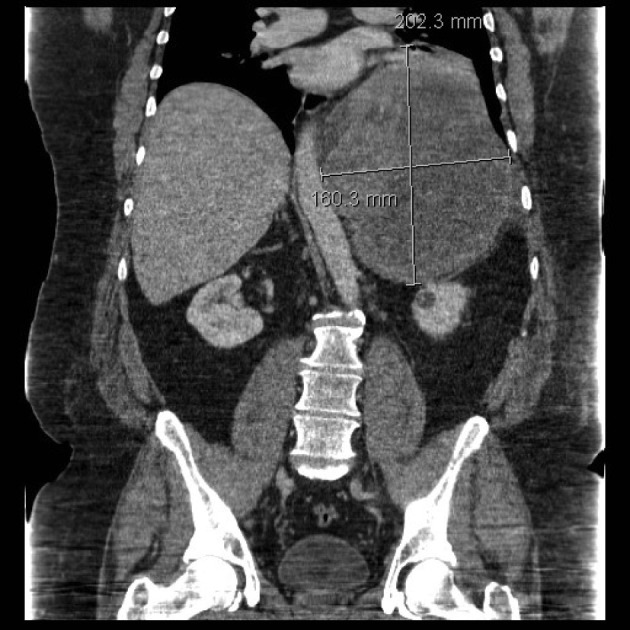
CT scan showing posterior mediastinal mass.

**Figure 2:**
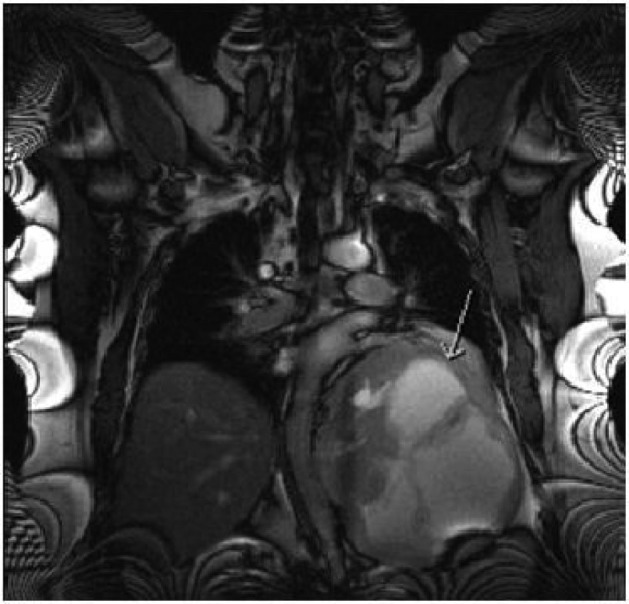
MRI features of the mass (arrow).

A 47-year-old obese man with severe mid-back pain for 6-months was found to have a large intra-thoracic mass on a routine plain film of the chest. A computed topography (CT) scan of the chest/abdomen/pelvis revealed a 20.5 × 15.5 × 16.0 cm heterogeneous mass in the left posterior mediastinum with effacement of the left lower lobe, left inferior pulmonary vein, displacement of hemi-diaphragm inferiorly and mediastinal structures towards to the right chest (Fig. 1). A magnetic resonance imaging (MRI) study revealed peripheral enhancement and internal necrosis (Fig. 2). CT-guided biopsy revealed a spindle cell tumor. Surgical resection was recommended.

**Figure 3:**
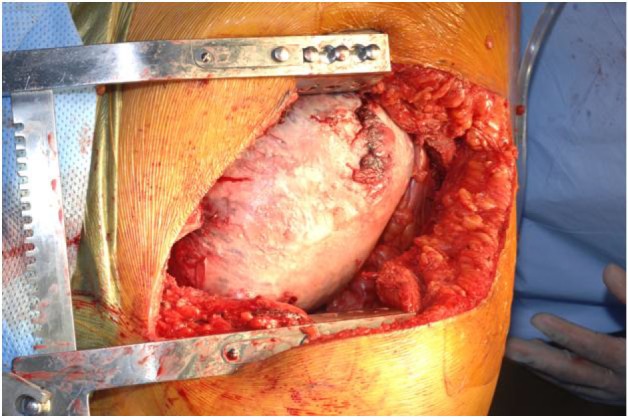
Tumor in-situ.

**Figure 4:**
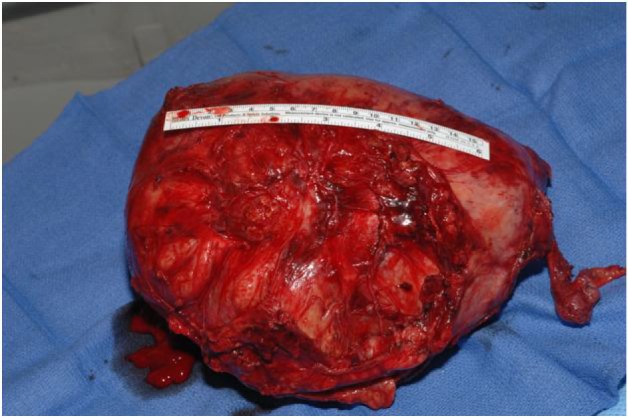
Resected mass.

A left thoraco-abdominal incision was made, extending along the 8th inter-space along the thorax (Fig. 3). Exposure was enhanced by resection of the 9th rib. The tumor abutted the descending aorta, and was adhered to the left lower lobe of the lung posteriorly as well as the adjacent diaphragm. Resection required en bloc wedge resection of a portion of adhered left lower lobe and the hemi-diaphragm. An extra-pleural dissection was undertaken to ensure adequate chest wall margins and along the thoracic aorta, a subadventitial resection was performed. The tumor appeared to originate from the nerve root in the area of 10th thoracic vertebra (Fig. 4). Frozen section of the pleural margin was found to contain no tumor. The diaphragmatic defect was primarily repaired.

**Figure 5:**
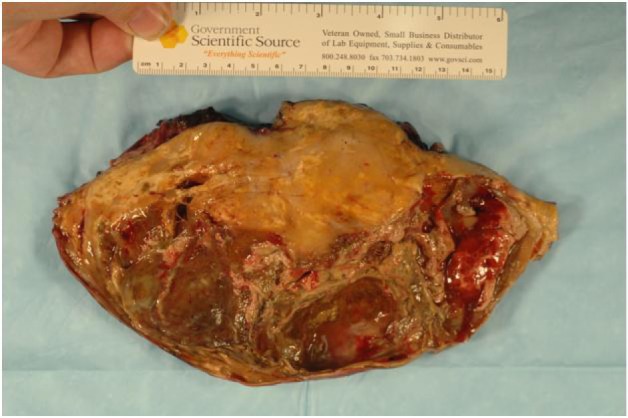
Cross-section of the mass.

**Figure 6:**
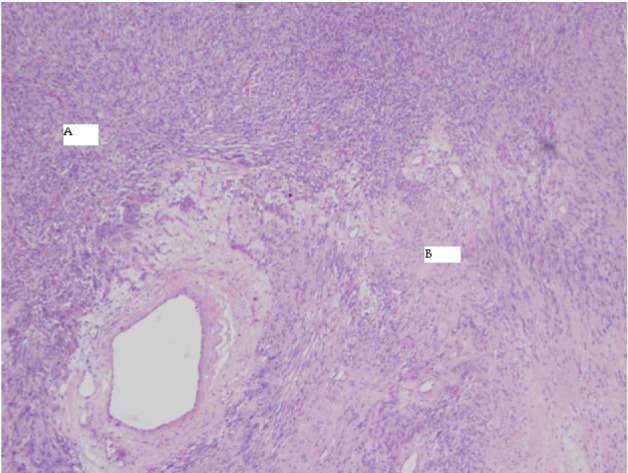
4X HE Antoni A and B.

**Figure 7:**
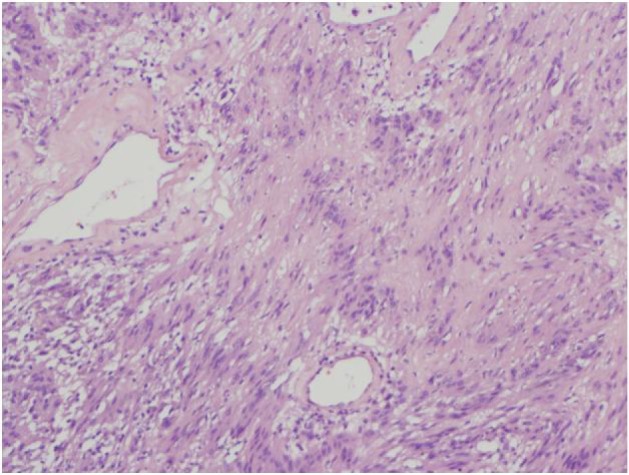
4X HE varocay.

**Figure 8:**
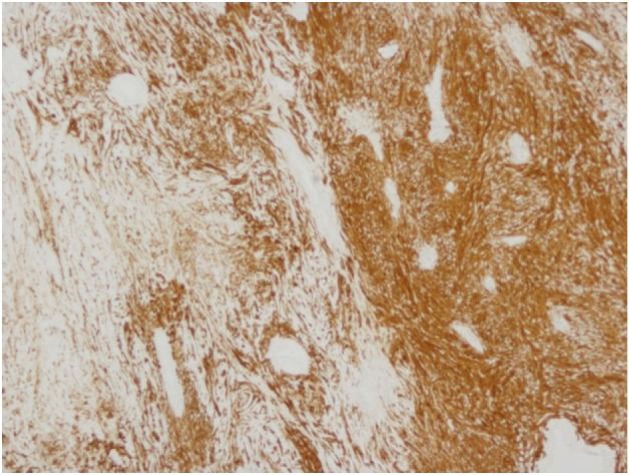
4X S100 Antoni A and B.

The final pathology demonstrated a 23 × 14.5 × 13.5 cm, 2590 grams, multiloculated, cystic mass (Fig. 4, 5) consistent with ancient schwannoma characterized by a proliferation of spindle cells (Fig. 6), Antoni type A and type B (Fig. 7) and strong positivity for S-100 protein (Fig. 8). The post-operative period was uneventful and patient was discharged home day 5 after surgery. At 11-month follow-up, the patient is free from recurrence.

## Discussion

Neurogenic tumors represent about 12-39% of all mediastinal tumors [2], 90% occur in the posterior mediastinum [3] and about 95% of these tumors are schwannomas [4]. Other tumors in the posterior mediastinum include parasitic cyst, meningocele, neurenteric cyst, gastroenteric cyst, thoracic duct cyst, primary esophageal tumors, and lymphoma [5]. Posterior mediastinal schwannomas can originate from spinal, paravertebral sympathetic, vagus or phrenic nerves and thoracic nerve roots [2, 6]. They are slow growing, with very low risk of malignant transformation [7]. Grossly, schwannomas are encapsulated, heterogeneous tumors with cystic degeneration [8]. Dumb-bell tumors occurs when paraspinal shwannomas extend through the intervertebral opening into the thoracic cavity.

Schwannomas occur equally between the sexes, and presentation is typically in patients older than 40 [6]. Most patients are asymptomatic and presentation with severe back pain is extremely rare. Large mediastinal tumors are often first identified incidentally by plain radiographs with follow up imaging such as CT scans showing a sharply demarcated mass with low densities, mild enhancement, and punctate calcification [9]. MRI remains the best diagnostic tool which shows hypodensity on T1-weighted images and hyperdensity on T2-weighted images [9].

Microscopically, the tumor contains varying proportions of Antoni A and B histologies [10]. Antoni-A is composed of spindle cells with elongated nuclei, forming interlacing bundles with focal nuclear palisading and lacks mitotic figures [11]. However, Antoni-B is less cellular and lacks distinct architectural features [10, 11]. Strong positively for S-100 is a requisite for diagnosis [11]. Since fine needle aspiration or biopsy cannot provide a definitive histological diagnosis [8], surgical resection is typically recommended to confirm diagnosis, obtain local control and mitigate symptoms when present.

Posterolateral thoracotomy has traditionally been the preferred surgical approach for resection of large posterior mediastinal tumors [8]. Video assisted thoracic surgery is ideal for smaller lesions and those free from vitals structures [12]. A tumor showing intraspinal extension (dumb-bell tumor) requires a combined neurosurgical and thoracic approach [13]. A clamshell approach has been reported in the management of anterior tumors due to size [14]. This case describes, one of the largest ancient schwannomas reported to date with intimate involvement with the diaphragm, left lower lung lobe and anterior surface of the descending thoracic aorta. With a thoracoabdominal approach, the operative exposure was excellent and allowed complete and safe resection of the tumor. This unique case shows that the operative strategies for giant posterior mediastinal tumors should be tailored to individual patients.

The tumor was confirmed to be an ancient schwannoma based on unique histopathologic features of fibrous background with spindle cells, strong positivity for S-100 and negative for neurofilament protein, and epithelial membrane antigen [7]. The specimen lacked atypia, and pleomorphism (Fig. 1, 2) hence a benign tumor [7] despite its massive size.

In conclusion, posterior mediastinal schwannomas are benign and usually asymptomatic but can grow to a large size causing significant compressive symptoms. Such massive size may require modification of the traditional posterolateral thoracotomy for safe resection. A thoraco-abdominal approach is ideal for resection of giant posterior mediastinal schwannomas or other tumors and is safe and may be considered in the management of these tumors.
